# Infantile nystagmus without overt eye abnormality: Early features and neuro‐ophthalmological diagnosis

**DOI:** 10.1111/dmcn.15284

**Published:** 2022-05-29

**Authors:** Agnese Suppiej, Chiara Ceccato, Valentina Lonardi, Maria E. Reffo

**Affiliations:** ^1^ Department of Medical Sciences, Paediatric Section University of Ferrara Ferrara Italy; ^2^ Robert Hollman Foundation Padova Italy

## Abstract

**Aim:**

To analyse the neuro‐ophthalmological data of children referred for further work‐up of infantile nystagmus where ophthalmological evaluation had not achieved a diagnosis.

**Method:**

We retrospectively reviewed medical records of patients presenting with infantile nystagmus at our institution between 2007 and 2019. Inclusion criteria were onset before 6 months of age, availability of complete ophthalmic examination, visual electrophysiological tests, and neurological examination. Children with a previous definite ophthalmological diagnosis at onset and those with uncertain nystagmus onset age were not recruited.

**Results:**

Out of 142 infants (mean age at nystagmus onset 3.6 mo, SD 1.7, range 0–6 mo; 56 females, 86 males), 23% had neurological nystagmus, 7% mixed neurological and sensory nystagmus, 48% sensory defect, and 22% idiopathic infantile nystagmus. The neurological diagnoses were inborn errors of metabolism, white matter genetic disorders, and brain malformations. The prevalent diagnosis in the sensory defect subgroup was retinal dystrophy.

**Interpretation:**

Infantile nystagmus without diagnostic ocular findings may be due to neurological, retinal, and optic nerve disorders or be a benign idiopathic condition. In infants with and without neurological abnormalities, the search for a sensory defect should include visual electrophysiology performed early in the diagnostic pathway.

**What this paper adds:**

- Infantile nystagmus without diagnostic ophthalmological signs has an underlying neurological cause in 30% of cases.
- Neurological diagnoses include congenital brain malformations, and metabolic and genetic disorders.
- Sensory defects are part of systemic neurological disorders in 23% of infants.
- Electrophysiology is useful when ophthalmological examination is uninformative.

## What this paper adds

- Infantile nystagmus without diagnostic ophthalmological signs has an underlying neurological cause in 30% of cases.
- Neurological diagnoses include congenital brain malformations, and metabolic and genetic disorders.
- Sensory defects are part of systemic neurological disorders in 23% of infants.
- Electrophysiology is useful when ophthalmological examination is uninformative.

**Abbreviations**
IIN: Idiopathic infantile nystagmus
MNSN: Mixed neurological and sensory nystagmus
OCT: Optical coherence tomography
SDN: Sensory defect nystagmus
VEP: Visual evoked potential

Nystagmus was defined by the child neurologist Jean Aicardì in his seminal book of paediatric neurology as ‘an involuntary, rhythmical, conjugate oscillatory movement of the eyes, due to dysfunction of the complex mechanisms that maintain ocular fixation’.^1^ The fixation reflex that maintains the visual target centred in the fovea requires fronto‐nigral‐collicular circuits and depends upon the motion detection (magnocellular) portion of the visual system.^2^ Early in life these anatomo‐functional systems are in their crucial developmental period; as these are driven by sensory input, they are consequently vulnerable to prechiasmal visual disorders.

The pathophysiological mechanisms of infantile nystagmus include sensory defects (disorders of the eye and anterior visual pathways causing visual deficit), a variety of neurological syndromes disrupting oculomotor circuits and congenital idiopathic nystagmus, nowadays preferentially called idiopathic infantile nystagmus (IIN), when no underlying eye or neurological disorders can be detected.^3^, ^4^ Because of the multifaceted pathophysiology of nystagmus, the clinical evaluation should include a close collaboration between the child neurologist, the ophthalmologist, and other related professionals using an interdisciplinary approach.

In the paediatric population, nystagmus with infantile onset before the age of 6 months, is the most common subtype and is also called early‐onset nystagmus. Nash et al. reported on a retrospective population‐based study of childhood nystagmus in Minnesota, they found nystagmus had an infantile onset in up to 87.3% of the cases, giving a birth prevalence of 12.1 per 10 000.^5^ In a population‐based study of infantile nystagmus in the Capital Region of Denmark, Hvid et al. reported a birth prevalence of 6.1 per 10 000.^6^ Given the difference, both studies stated that ocular diseases are the most common cause of infantile nystagmus, in 32.4% and 44% of cases respectively.^5^, ^6^

Infants with sensory defects due to ocular malformations, anterior segment disorders, or evident retinal pathologies can be easily identified soon after birth by eye inspection and fundus oculi examination that drive subsequent instrumental work‐up, leading to differential diagnosis and treatment decision. Conversely, when the above ocular abnormalities have been ruled out, or when fundus oculi show mild and non‐specific features, electroretinogram and visual evoked potentials (VEPs) should be an essential part of the investigative pathway of the infant presenting with abnormal eye movements and poor vision. Visual electrophysiology is able to highlight visual function abnormalities even in the absence of fundus oculi changes;^7^, ^8^, ^9^ it requires dedicated methodology, special expertise, and age‐related normative data. This also applies to other highly specialized instruments for neuro‐ophthalmological diagnosis such as spectral‐domain optical coherence tomography (OCT).^10^ Moreover, the diagnostic pathway of infants with nystagmus without overt eye abnormalities should include a thorough neurological work‐up aimed at ruling out those neurological disorders affecting the oculomotor system.

At our institution we have a well‐established electrophysiological assessment process based on international guidelines^11^ adapted to young children.^12^, ^13^ It is used to complement the neuro‐ophthalmological evaluation of infants with nystagmus. The purpose of this study was to evaluate a cohort of children referred for further work‐up of infantile nystagmus where ophthalmological evaluation had not so far achieved a diagnosis. The ultimate goal was to characterize, in this special population, the neuro‐ophthalmological features in early infancy with respect to pathophysiological categorization and final interdisciplinary diagnosis.

## METHOD

### Design and procedures

In the present study we retrospectively reviewed the records of children with early‐onset nystagmus admitted to our institution from 1st January 2007 to 15th December 2019.

We used patient records to extract data on ophthalmological and neurological examination, brain magnetic resonance imaging (MRI), OCT, visual electrophysiology (at onset and at follow‐up), metabolic and genetic panels, and the final diagnosis. The standard methodology for visual electrophysiology in our institution was the same throughout the study period.^12^, ^13^, ^14^

The retrospective analysis of data was approved by the Institutional Board of the Robert Hollman Foundation (N. R05/2017). Data collection and analysis adhered to the Declaration of Helsinki for research involving human participants.

### Participants

The inclusion criteria were: (1) onset of nystagmus before 6 months of age clearly stated; (2) minimum data set of family and personal history, fundus oculi, and neurological examination performed close to the clinical onset; (3) electroretinogram and VEPs performed at a time point close to clinical onset; (4) at least one clinical and electrophysiological follow‐up performed after 1 year from the clinical onset. The exclusion criterion was a definite ophthalmological diagnosis at the first ophthalmological visit.

Out of 189 infants meeting the inclusion criteria, 47 were excluded because of ocular malformations and preretinal disorders (*n*=21), retinopathy of prematurity (*n*=12) and oculocutaneous albinism (*n*=14) (Figure S1a).

### Infantile nystagmus classification

Based on all the available neuro‐ophthalmological and electrophysiological characteristics, we classified patients according to Casteels et al.^3^ Sensory defect nystagmus (SDN) was defined by the diagnosis of retinal or anterior visual pathway disorders; retinal dystrophies were further distinguished by subtypes as previously reported.^13^ Neurological nystagmus was defined by clinical and/or MRI abnormalities pointing to neurological disorders involving centres regulating eye movements. IIN was diagnosed in patients in whom SDN and neurological nystagmus were ruled out. We added to the Casteels et al. classification a fourth category accounting for children with a sensory defect within a primary neurological diagnosis. We called this new category mixed neurological and sensory nystagmus (MNSN). The neuro‐ophthalmological algorithm used for infantile nystagmus classification is summarized in Figure S1b.

The neuro‐ophthalmological and electrophysiological profiles were analysed in the above four diagnostic subgroups.

## RESULTS

The final study population included 142 patients (56 females and 86 males) with a mean age at nystagmus onset of 3.6 months (SD 1.7, range 0–6 mo). All patients had fundus oculi, electroretinogram, VEPs, neurological examination, and family history data. Of the 142 infants, 87 (61%) had performed MRI, 17 (12%) OCT, 45 (32%) genetic testing, and 13 (9%) full metabolic work‐up (Table 1).

**Table 1:** Diagnostic exams performed per nystagmus subtype

			SDN (*n*=68)				
	NN (*n*=33)	MNSN (*n*=10)	Retinal dystrophies (*n*=51)	Optic nerve disorders (*n*=7)	Ocular albinism (*n*=5)	Foveal hypoplasia (*n*=5)	IIN (*n*=31)
ERG	33 (100)	10 (100)	51 (100)	7 (100)	5 (100)	5 (100)	31 (100)
VEPs	33 (100)	10 (100)	51 (100)	7 (100)	5 (100)	5 (100)	31 (100)
OCT	0	0	9 (18)	0	0	5 (100)	2 (6)
Brain MRI	33 (100)	10 (100)	28 (55)	5 (71)	0	2 (40)	9 (30)
Neuropaediatric assessment	33 (100)	10 (100)	0	0	0	0	0
Genetic testing	2 (6)	2 (6)	31 (61)	0	1 (20)	3 (60)	1 (3)
Metabolic work‐up	8 (24)	5 (50)	0	0	0	0	0

Data are *n* (%). Abbreviations: NN, neurological nystagmus; MNSN, mixed neurological and sensory nystagmus: SDN, sensory defect nystagmus: IIN, idiopathic infantile nystagmus; ERG, electroretinogram; VEPs, visual evoked potentials; OCT, optical coherence tomography; MRI, magnetic resonance imaging.

The number of patients in the different nystagmus subtypes is reported in Figure S1.

### Neurological nystagmus

**Figure 1:**
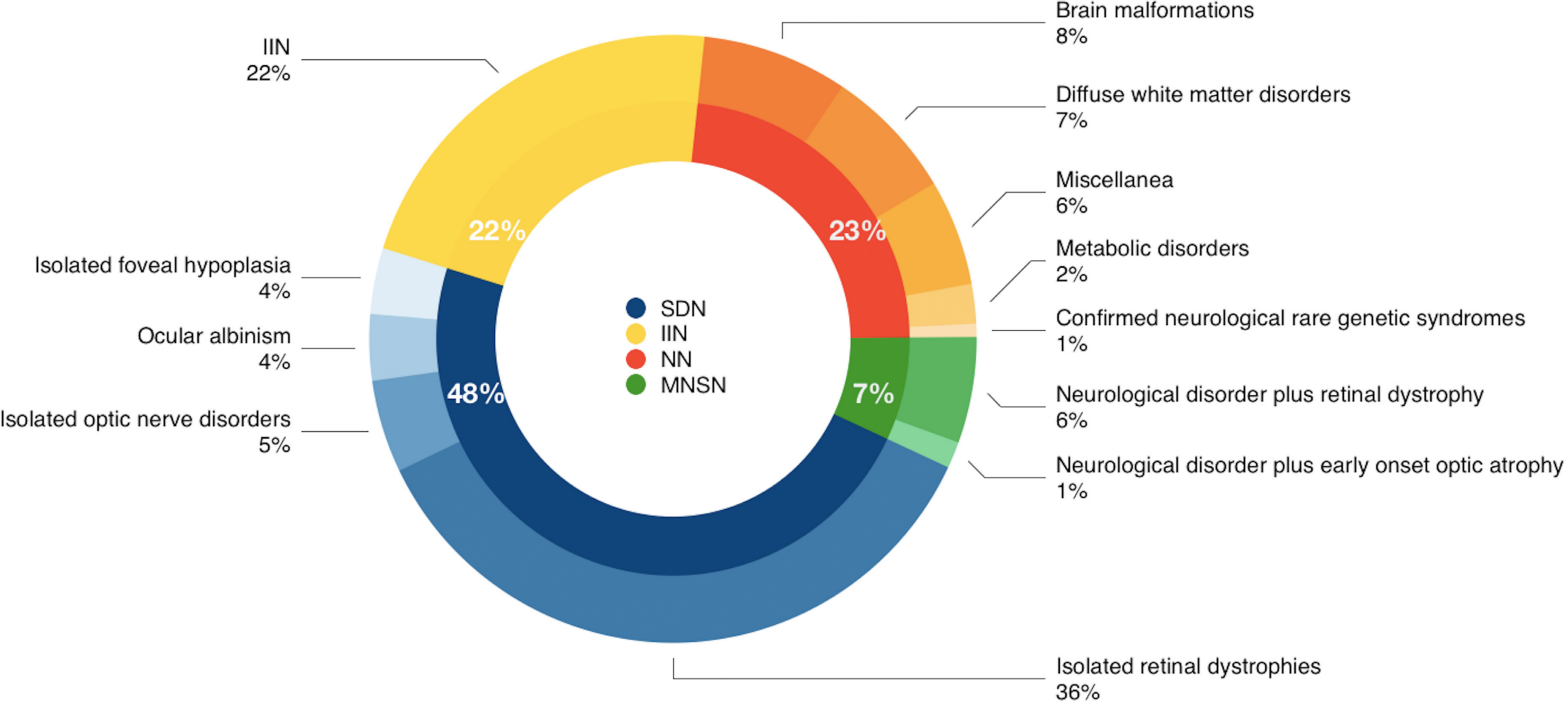
Infantile nystagmus subtype classification (internal circle). Clinical diagnosis for each subgroup expressed as percentage of the total population (external circle). Abbreviations: IIN, idiopathic infantile nystagmus; MNSN, mixed neurological and sensory nystagmus; NN, neurological nystagmus; SDN, sensory defect nystagmus.

Of the 142 patients, 33 (23%) were included in the subgroup with neurological nystagmus; the neurological diagnoses are summarized in Figure 1. They included congenital malformations of posterior cranial fossa structures and their supratentorial connections, diffuse white matter disorders such as early‐onset leukodystrophies, diffuse brain involvement in rare genetic syndromes, congenital disorders of metabolism. Moreover, there was a heterogenous group of neurodevelopmental disorders with or without epilepsy in whom the etiological diagnosis was not available (miscellanea). Details of the diagnoses can be found in Table S1. The neurological examination performed within 6 months from nystagmus onset was abnormal in all infants except two patients (patient 13 and patient 15 see Table S1), both having transient isolated pontine tegmental hyperintensity at MRI,^15^ and substantially normal neurological examination at 1 year and 6 years follow‐up. The brain MRI was abnormal in all remaining cases.

### Mixed neurological and sensory nystagmus

Ten patients had a SDN within a primary neurological diagnosis (7%) and were classified as MNSN. In these patients the pathophysiology of nystagmus was a mixed type due to both oculomotor circuits involvement and sensory defect. The diagnosis of eight out of these ten infants all affected by retinal dystrophy included: three methylmalonic aciduria with homocystinuria, two mitochondrial disorders, one Galloway‐Mowat syndrome, one Joubert syndrome, and one tricotiodystrophy. In the remaining two infants with epileptic encephalopathy associated to 3p26.3 duplication and pericentric inversion chromosome 8 syndromes, the sensory defect was due to early‐onset optic atrophy (Table S1).

### Sensory defect nystagmus

Of the 142 patients, 68 (48%) were classified in the subgroup with SDN. The ophthalmological diagnoses are summarized in Figure 1.

At the first level ophthalmological evaluation, 40 out of 68 (59%) infants had completely normal fundus oculi while the remaining 28 patients had mild non‐specific changes not considered as pathological. Common findings in these patients were mild pigmentary changes or hypopigmentation, doubtful alteration of the foveal reflex, slightly pale optic disk, all difficult to distinguish from physiological immaturity.

All except 13 infants (81%) had a normal neurological examination and no delay in acquisition of motor milestones (sitting, walking). The remaining children (19%) had a normal neurological examination but showed delay in achieving sitting and walking. All attained independent walking between 18 months and 20 months, eight infants after a short physiotherapy cycle.

The MRI performed in 51% of the cases (35/68) was normal in all except for small optic nerves in patients affected by optic nerve disorders. OCT scans were available in 21% (14/68) of infants, only at follow‐up at a mean age of 10 years (range 3–13 y).

The aetiology of SDN was isolated retinal dystrophy in 51 out of 68 patients (75%). The retinal subtypes were: 22 out of 51 achromatopsia, 9 out of 51 Leber congenital amaurosis defined as early‐onset rod‐cone dystrophy with flat electroretinogram and VEPs, 4 out of 51 severe congenital early‐onset retinal dystrophy defined as early‐onset rod‐cone dystrophy showing residual visual function in the first years of life, 5 out of 51 rod‐cone dystrophy, 5 out of 51 *CACNA1F*‐related congenital stationary night blindness, 4 out of 51 cone‐rod dystrophy, 1 out of 51 congenital stationary night blindness, and 1 out of 51 an atypical form of cone dystrophy. The electroretinogram was abnormal in all patients, with distinctive patterns of abnormalities. Genetic testing, performed in 31 out of 51 children (61%), identified genetic variants in genes causative of inherited retinal dystrophies in 27 out of 31 patients. In the remaining four, whole exome sequencing was negative. Of the remaining 17 patients with SDN, seven had optic nerve hypoplasia, five had ocular albinism, and five had isolated foveal hypoplasia.

In optic nerve hypoplasia, the optic disk was described as normal in two out of seven patients and slightly pale in the remaining five. All had poor visual behaviour, abnormal VEPs, but normal electroretinogram. Five had reduced optic nerve diameter at MRI and two both reduced optic nerve and chiasma size at MRI.

In ocular albinism infants’ skin and hair were pigmented. The fundus oculi showed in all infants mild nonspecific albinoid‐like findings at the first visit. The characteristic crossed asymmetry pattern was demonstrated by VEPs in all except one. This patient had an atypical crossed asymmetry pattern and at whole exome analysis he was found carrier of the p.Gln680Ter(c.2038C>T) and p‐Leu22ArgfsTer33 (c.60_64dup) pathogenic variants in heterozygosis in the *HPS6* gene allowing the diagnosis of Hermansky‐Pudlak syndrome.

Isolated foveal hypoplasia was diagnosed in five infants by means of OCT; all were previously classified as IIN. All but one showed at follow‐up mild pigmentary changes associated or not to mild foveal reflex alteration at fundus oculi examination. Whole exome analysis performed in three out of five patients, all siblings, found pathogenic variants in the *GPR143* gene (p. Leu258Ter (c.773T>A)).

### Idiopathic infantile nystagmus

Thirty‐one infants were diagnosed as IIN (22%). At nystagmus onset all had normal neurological examination, normal MRI (performed in nine cases), and normal electroretinogram. None had visual findings other than nystagmus. The fundus oculi was normal in all cases. The VEPs were abnormal in 15 cases but all normalized at a median age of 27 months (range 8–76 mo) showing a transient delay in the maturation of cortical visual responses.

The OCT, performed in two children, was normal. One patient underwent whole exome sequencing but no pathogenetic variants were found.

## DISCUSSION

Nystagmus is a worrying condition for the family and paediatricians. In child neurology, it may indicate severe intracranial pathology even in the absence of other neurological signs. However, in infancy the most frequent aetiology of nystagmus is eye or anterior visual pathways disorder allowing for SDN.^5^, ^6^ Infantile nystagmus without major ocular signs thus create a diagnostic challenge for the child neurologist.

In the present study we collected a large cohort of children with infantile nystagmus uniquely characterized by the absence of pathognomonic ocular abnormalities and systematically evaluated with an integrated interdisciplinary neuro‐ophthalmological approach including visual electrophysiology. Clinical, ophthalmological, and neurological examinations combined with electrophysiological assessments (electroretinogram‐VEPs) and, in selected cases, full neurodiagnostic work‐up enabled classification of all our patients into one of the four diagnostic subgroups: neurological nystagmus, MNSN, SDN, IIN.

Our data confirmed that SDN is the most prevalent type of infantile nystagmus even in children without obvious ocular diagnoses, but about one‐third of the infants in our cohort had a variety of neurological malformations and genetic disorders involving oculomotor circuits, as confirmed by MRI, manifesting early in life with nystagmus. We demonstrated that paediatric neurological nystagmus is not only due to ‘acquired’ brain damage, usually occurring in older children,^16^ but also to genetic disorders and brain malformations manifesting with infantile nystagmus. In agreement with recent literature,^17^ neurological pathophysiology should be considered in the diagnostic work‐up of infantile nystagmus. Nystagmus may also be idiopathic in a minor proportion of infants, further complicating aetiological work‐up.

Vision loss can impact on psychomotor development;^18^ thus the evaluation of the infant’s level of vision coupled with basic neurological assessment is needed to assist clinicians in teasing apart the developmental consequences of visual impairment from neurological abnormality.

Considering the neurological diagnoses associated with neurological nystagmus and MNSN, white matter, metabolic diseases, and cerebral malformations were the most prevalent disorders. The neurological examination was clearly abnormal in all but two infants both showing isolated nonprogressive pontine tegmental hyperintensities on MRI. Considering children with isolated SDN, none had clearly abnormal neurological signs; however, we observed transient developmental delay of gross motor skills resolving over time in some children with severe vision loss, attributed to the impact of low vision. From these results we suggest that nystagmus presenting early in life, and associated with abnormalities in the neurological examination, should undergo a complete neurological diagnostic work‐up including MRI. At this early age, neurological signs of fronto‐cerebellar involvement other than nystagmus may be non‐specific (i.e. hypotonia instead of coordination disorder in cerebellar abnormalities) and may be missed on clinical grounds alone. MRI and a full neurometabolic and genetic work‐up are fundamental to achieve the diagnosis in these children. Electrophysiology should be performed before MRI in infants without neurological signs, in contrast with the practice reported by Bertsch et al.^17^ The authors showed that, in infantile nystagmus, MRI was usually performed as the first instrumental test.

One other important finding of the present study was that 23% of the total number of infants with a primary neurological diagnosis had an associated sensory defect, classified as MNSN. These infants had a mixed pathophysiology of nystagmus involving both the oculomotor circuits function and the sensory systems such as the retina or optic nerve. Examples were the occurrence of rod‐cone retinal dystrophy in some metabolic diseases and in Joubert syndrome, Leber congenital amaurosis in Galloway‐Mowat syndrome, and early‐onset optic atrophy in genetic syndromes. Vision impairment can be overlooked in such cases because of the predominance of the neurological features overshadowing abnormal visual clues.^19^ Electrophysiological tests can be of paramount importance in the identification of the associated retina and anterior visual pathways disorders, representing diagnostic clues in the syndromic definition.

Visual electrophysiology had an important role also in the subgroup with sensory defect, in line with research.^20^, ^21^ The electroretinogram identified photoreceptors’ dysfunction in all retinal dystrophies, giving different patterns of involvement, useful for genotype–phenotype correlations.^13^ The VEPs supported the diagnosis of ocular albinism in infants without cutaneous findings, by identifying misrouting of optic nerve fibres, the so‐called pattern of crossed asymmetry,^22^ provided that the electroretinogram excluded a retinal dystrophy.^23^ Moreover, recent genetic advances report that, in the absence of albino fundus, the crossed asymmetry pattern can suggest variations in the gene *SLC38A8*.^24^

The subgroup with SDN also included children with isolated foveal hypoplasia, in agreement with other studies.^25^, ^26^ In infancy, these patients fulfilled our diagnostic criteria for IIN, but foveal hypoplasia was subsequently diagnosed by means of OCT, at a median age of 8 years, thanks to long‐term follow‐up. Whole exome sequencing revealed *GPR143* variants in three male siblings with foveal hypoplasia. The ophthalmological diagnosis of foveal hypoplasia may be difficult at fundus oculi inspection alone in very young infants due to retinal immaturity, abnormal ocular movements, and scarce cooperation. In infancy, the OCT technique is also not easy to perform and less reliable, explaining the late diagnosis in these patients.

In recent years, new knowledge about OCT technology^27^ combined with next‐generation sequencing whole exome analysis made it possible to understand that some children previously diagnosed with IIN may be affected by genetic retinal disorders. *FRMD7*, *GPR143*, and *SLC38A8* are the genes most frequently reported in literature.^28^, ^29^, ^30^

With regard to the role of VEPs abnormalities, they were seen in some of our patients with optic nerve or brain disorders affecting the visual pathways and consisted of delayed latency and/or reduced amplitude. Considering that VEPs represent the visual cortex activation after a visual stimulus, but they do not localize the pathological site, the diagnosis of optic nerve hypoplasia was supported by a combination of VEPs and MRI focused on the optic nerves.

IIN was the rarest subtype in our cohort, accounting for 22% of the cases. It was prevalent in males (74%). The diagnosis of these patients was that of exclusion, supported by normal neurological and ophthalmological work‐up. VEPs were the only test transiently abnormal in 48%, suggesting a delay in VEPs maturation consequent to eye movement effect per se.

Limitations of this study included its retrospective nature which reduced the availability of comprehensive diagnostic data and precluded group comparisons, since data were not collected at the same age.

In conclusion, infantile nystagmus in the absence of ophthalmological signs is subtended by a variety of ophthalmological and neurological disorders that require an interdisciplinary neuro‐ ophthalmological approach. We propose that electrophysiological testing could be performed early in the diagnostic pathway of these infants, in order to rule out retinal or optic nerve disorders both in children with and without neurological signs or symptoms. Brain MRI and a full neurometabolic and/or genetic work‐up should be first considered in infants with abnormal neurological examination or developmental delay. When the neurological examination is fully normal, psychomotor development is appropriate for age, and the electroretinogram and VEPs are normal, the diagnostic hypothesis of IIN should be confirmed at follow‐up when fundus oculi evaluation may be more reliable, and OCT can further support a possible diagnosis of foveal hypoplasia.

## Supporting information


**Table S1:** Neurological diagnoses

**Figure S1:** Flow diagram of the study recruitment and neuro‐ophthalmologic algorithm used for infantile nystagmus classification

## Data Availability

Data available on request from the authors.
