# Supplementary figures and images for: Infantile nystagmus without overt eye abnormality: Early features and neuro‐ophthalmological diagnosis
Source: Dev Med Child Neurol. 2022 May 29;64(12):1532–8. doi: 10.1111/dmcn.15284 (PMC9796881; doi:10.1111/dmcn.15284)

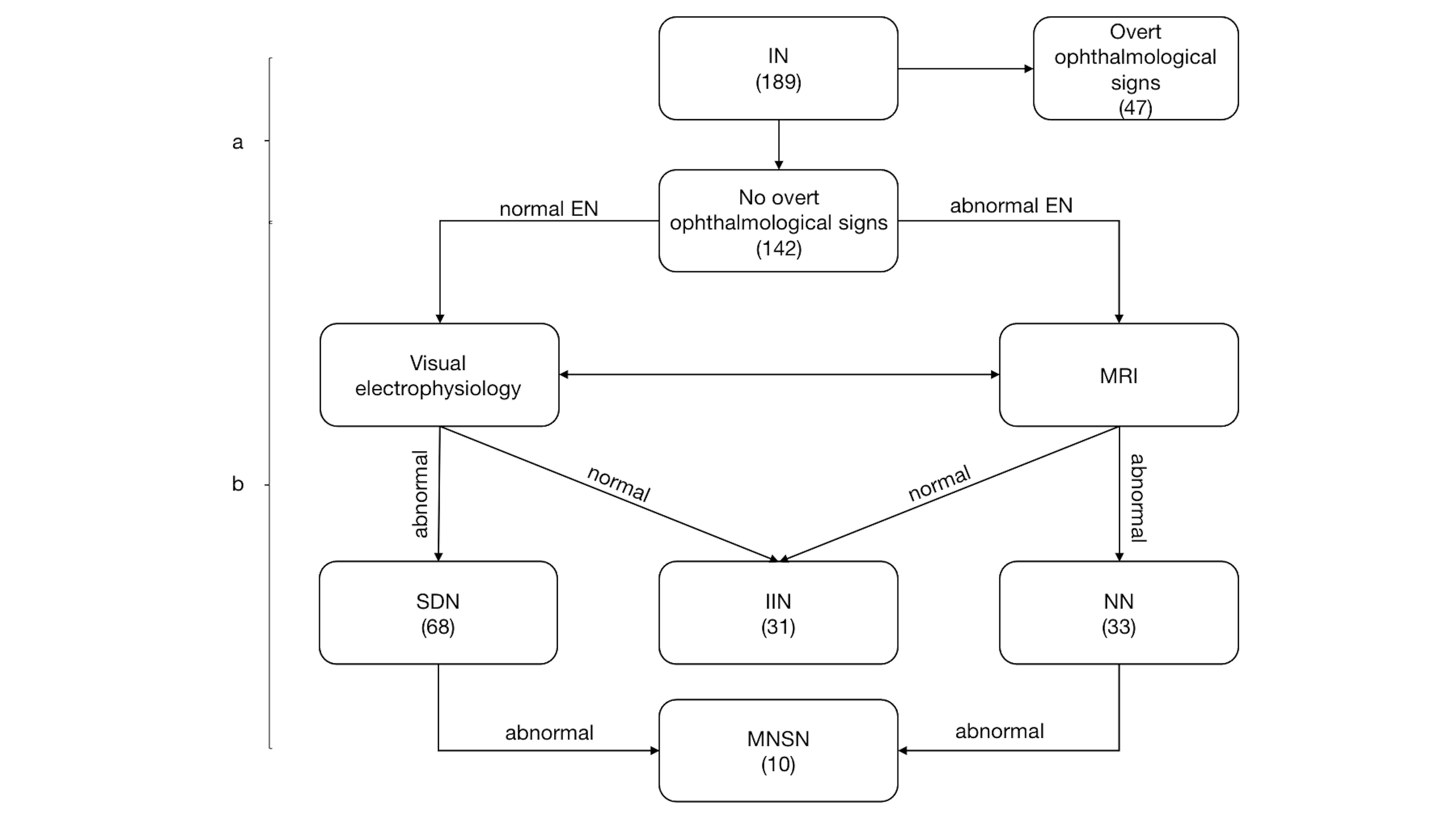

Supplement: Supplementary file 1 — Table S1: Neurological diagnoses [file DMCN-64-1532-s001.png]
