# Supplementary material for: Infantile nystagmus without overt eye abnormality: Early features and neuro‐ophthalmological diagnosis
Source: Dev Med Child Neurol. 2022 May 29;64(12):1532–8. doi: 10.1111/dmcn.15284 (PMC9796881; doi:10.1111/dmcn.15284)
Supplement: Supplementary file 2 — Figure S1: Flow diagram of the study recruitment and neuro‐ophthalmologic algorithm used for infantile nystagmus classification [file DMCN-64-1532-s002.pdf]

ID	Group disorders	Neurological Diagnosis	Sensory Defect	MRI oculomotor system involvement
1	Brain malformation	Joubert syndrome	-	+
2	Brain malformation	Complex posterior cranial fossa malformation	-	+
3	Brain malformation	Arnold Chiari type 1 malformation	-	+
4	Brain malformation	Cerebellar hypoplasia and corpus callosum dysgenesis	-	+
5	Brain malformation	Dysmorphic brainstem and corpus callosum hypoplasia	-	+
6	Brain malformation	Pontine tegmental cap dysplasia	-	+
7	Brain malformation	Complex brain malformation involving brainstem, corpus callosum, caudatum and lenticular nuclei, silvian fissure	-	+
8	Brain malformation	Cortical malformation with frontotemporal prevalence and cerebellar vermis hypoplasia	-	+
9	Brain malformation	Prenatal torcular of Erofili trombosis with extensive brain damage	-	+
10	Brain malformation	Galloway Mowat syndrome	Leber's Congenital Amaurosis	+
11	Brain malformation	Joubert syndrome	Rod-cone dystrophy	+
12	Brain malformation	Schizencephaly and areas of cortical heterotopia	-	
13	Miscellanea	Isolated pontine tegmental hyperintensity	-	+
14	Miscellanea	Trichothiodystrophy	Rod-cone dystrophy	+
15	Miscellanea	Isolated pontine tegmental hyperintensity	-	+
16	Brain malformation	Dysmorphic brainstem and cerebellar vermis hypoplasia	-	+
17	Methabolic disorder	Leigh syndrome	-	+
18	Methabolic disorder	Leigh syndrome	-	+
19	Miscellanea	Progressive epileptic encephalopaty ndd	-	+
20	Methabolic disorder	Primary coenzime Q10 defiency	Rod-cone dystrophy	+
21	Methabolic disorder	Congenital defect of glycosylation	SECORD	+
22	Methabolic disorder	Respiratory chain mitochondrial disorder	-	+
23	Methabolic disorder	Methylmalonic aciduria homocystinuria	Rod-cone dystrophy	+
24	Methabolic disorder	Methylmalonic aciduria homocystinuria	Rod-cone dystrophy	

25	Methabolic disorder	Methylmalonic aciduria homocystinuria	Rod-cone dystrophy	+
26	Diffuse white matter disorder	Pelitzaeus Merzbacher disease	-	+
27	Diffuse white matter disorder	Pelitzaeus Merzbacher-like disease	-	+
28	Diffuse white matter disorder	Intellectual disability and diffuse white matter abnormality	-	+
29	Diffuse white matter disorder	Pelitzaeus Merzbacher-like disease	-	+
30	Diffuse white matter disorder	Pelitzaeus Merzbacher-like disease	-	+
31	Diffuse white matter disorder	Cortical malformation and central hypomyelination	-	+
32	Diffuse white matter disorder	Central hypomyelination	-	+
33	Diffuse white matter disorder	Pelitzaeus Merzbacher disease	-	+
34	Diffuse white matter disorder	Pelitzaeus Merzbacher disease	-	+
35	Diffuse white matter disorder	Central hypomyelination	-	+
36	Confirmed diffuse brain involvement in rare genetic syndromes	3p26.3 duplication syndrome and epileptic encephalopathy	Early onset optic atrophy	
37	Confirmed diffuse brain involvement in rare genetic syndromes	Pericentric inversion chromosome 8 syndrome and epileptic encephalopathy	Early onset optic atrophy	
38	Miscellanea	Epileptic encephalopathy ndd	-	
39	Miscellanea	Epileptic encephalopathy ndd	-	
40	Miscellanea	Delayed myelination and macrocephaly	-	
41	Miscellanea	Epileptic encephalopathy ndd	-	
42	Miscellanea	Drug resistant epilepsy and cortical athrophy ndd	-	
43	Confirmed diffuse brain involvement in rare genetic syndromes	CDKL5 epileptic encephalopathy	-	
